# Supplementary material for: Getting More Out of Biomedical Documents with GATE's Full Lifecycle Open Source Text Analytics
Source: PLoS Comput Biol. 2013 Feb 7;9(2):e1002854. doi: 10.1371/journal.pcbi.1002854 (PMC3567135; doi:10.1371/journal.pcbi.1002854)
Supplement: Dataset S1 — GATE software. Dataset S1 bundled with this paper contains a distribution of GATE (or see http://gate.ac.uk/download/). (TGZ) [file pcbi.1002854.s001.tgz › plos-gate/plugins/Tagger_MetaMap/doc/gate/metamap/package-tree.html]

gate.metamap Class Hierarchy


---


|  |  |  |
| --- | --- | --- |
| **Overview** \| **Package** \| Class \| Use \| **Tree** \| **Deprecated** \| **Index** \| **Help** | |  |
| PREV   **NEXT** | **FRAMES**    **NO FRAMES**     **All Classes** |  |


---


## Hierarchy For Package gate.metamap

**Package Hierarchies:**: All Packages

---

## Class Hierarchy

- java.lang.Object
  - gate.util.AbstractFeatureBearer (implements gate.util.FeatureBearer, java.io.Serializable)
    - gate.creole.AbstractResource (implements gate.Resource, java.io.Serializable)
      - gate.creole.AbstractProcessingResource (implements gate.creole.ANNIEConstants, gate.ProcessingResource)
        - gate.creole.AbstractLanguageAnalyser (implements gate.LanguageAnalyser)
          - gate.metamap.**MetaMapPR** (implements gate.ProcessingResource, java.io.Serializable)

## Enum Hierarchy

- java.lang.Object
  - java.lang.Enum<E> (implements java.lang.Comparable<T>, java.io.Serializable)
    - gate.metamap.**OutputMode**- gate.metamap.**TaggerMode**

---


|  |  |  |
| --- | --- | --- |
| **Overview** \| **Package** \| Class \| Use \| **Tree** \| **Deprecated** \| **Index** \| **Help** | |  |
| PREV   **NEXT** | **FRAMES**    **NO FRAMES**     **All Classes** |  |


---
